# A novel homozygous *SLC39A14* variant in an infant with hypermanganesemia and a review of the literature

**DOI:** 10.3389/fped.2022.949651

**Published:** 2023-01-17

**Authors:** Meijiao Zhang, Liping Zhu, Huiping Wang, Ying Hao, Qingping Zhang, Chunyan Zhao, Xinhua Bao

**Affiliations:** ^1^ Department of Pediatrics, Peking University First Hospital, Beijing, China; ^2^ Department of Pediatrics, Linyi People's Hospital, Linyi, China; ^3^ Department of Neurology, Children's Hospital Affiliated to Kunming Medical University, Kunming, China; ^4^ Department of Pediatrics, Yuhuangding Hospital, Yantai, China

**Keywords:** hypermanganesemia, dystonia, spasticity, *SLC39A14*, HMNDYT2

## Abstract

**Background:**

Manganese (Mn) is an essential trace metal necessary for good health; however, excessive amounts in the body are neurotoxic. To date, three genes (*SLC30A10*, *SLC39A8*, and *SLC39A14*) have been discovered to cause inborn errors in Mn metabolism in humans. As very rare diseases, the clinical features require further clarification.

**Methods:**

A male Chinese patient who mainly presented with hypermanganesemia and progressive parkinsonism–dystonia was recruited for this study. We collected and analyzed clinical information, performed whole-exome sequencing (WES), and reviewed the relevant literature.

**Results:**

The motor-developmental milestones of the patient were delayed at the age of 4 months, followed by rapidly progressive dystonia. The patient displayed elevated Mn concentrations in blood and urine, and brain magnetic resonance imaging (MRI) showed symmetrical hyperintensity on T1-weighted images and hypointensity on T2-weighted images in multiple regions. A novel homozygous variant of the *SLC39A14* gene (c.1058T > G, p.L353R) was identified. The patient was treated with disodium calcium edetate chelation (Na_2_CaEDTA). Three months later, mild improvement in clinical manifestation, blood Mn levels, and brain MRI was observed. To date, 15 patients from 10 families have been reported with homozygous mutations of *SLC39A14*, with a mean age of onset of 14.9 months. The common initial symptom is motor regression or developmental milestone delay, with a disease course for nearly all patients involving development of progressive generalized dystonia and loss of ambulation before treatment. Additionally, hypermanganesemia manifests as Mn values ranging from 4- to 25-fold higher than normal baseline levels, along with brain MRI results similar to those observed in the recruited patient. Nine *SLC39A14* variants have been identified. Seven patients have been treated with Na_2_CaEDTA, and only one patient achieved obvious clinical improvement.

**Conclusion:**

We identified a novel *SLC39A14* mutation related to autosomal recessive hypermanganesemia with dystonia-2, which is a very rare disease. Patients present motor regression or delay of developmental milestones and develop progressive generalized dystonia. Chelation therapy with Na_2_CaEDTA appears to effectively chelate Mn and increase urinary Mn excretion in some cases; however, clinical response varies. The outcome of the disease was unsatisfactory. This study expands the genetic spectrum of this disease.

## Introduction

Manganese (Mn) is an essential trace metal required for normal growth and development in humans, acts as a cofactor for multiple enzymes, and plays a key role in metabolism, connective tissue formation, and immune function (1, 2). Mn is widely present in various foods, with a nutritional Mn deficiency rarely encountered (3–5). Excess Mn in the body is neurotoxic and can lead to the extrapyramidal syndrome by deposition in the brain, especially the basal ganglia (6). Mn toxicity can occur in miners, welders, individuals drinking contaminated water, and patients receiving prolonged total parenteral nutrition (7). Recently, Mn dyshomeostasis was associated with inherited genetic defects that involve genes encoding metal transporters (*SLC30A10*, *SLC39A8*, and *SLC39A14*) (8). Among them, *SLC39A14*-related manganism is the most recently discovered disorder named hypermanganesemia with dystonia type-2 (HMNDYT2; MIM 617013), which was first reported in 2016 (6). To date, 15 patients from 10 families have been reported with this disorder (6, 8–11).

In this study, we report a novel *SLC39A14* mutation identified in a 10-month-old boy in the Department of Pediatrics, Peking University First Hospital. Here, we present the clinical features of the patient and a review of the relevant literature.

## Methods

### Patient and clinical study

A 10-month-old Chinese boy mainly presenting with hypermanganesemia and progressive parkinsonism–dystonia was recruited. Clinical information was collected and analyzed, including disease onset, clinical manifestations, family history, auxiliary tests, and the outcome of chelation therapy with Na_2_CaEDTA, and the genomic DNA of the patient and his parents was extracted from peripheral leukocytes. Dystonia before and after treatment was scored by the Global Dystonia Rating Scale (GDS). Written informed consent was obtained from the parents. This study was approved by the Medical Ethics Committee at Peking University First Hospital (2022 research 077-001).

### Whole-exome sequencing (WES)

WES was performed by Beijing Joy Orient Translational Medicine Research Center Co., Ltd. (Beijing, China) using the SureSelect Human All Exon kit (v.6.0; Agilent Technologies, Santa Clara, CA, USA) and a Novaseq 6000 system (Illumina, San Diego, CA, USA). Sequence data were aligned to the UCSC hg19 reference genome (GRCh37) with the Burrows-Wheeler aligner (http://bio-bwa.sourceforge.net/). Aligned reads were processed with SAMtools (http://samtools.sourceforge.net/) and Picard (https://broadinstitute.github.io/picard/) according to the best practice guidelines of the Genome Analysis Tool Kit. Single-nucleotide variants and small insertion–deletions were detected with the Genome Analysis Tool Kit HaplotypeCaller (https://gatk.broadinstitute.org/hc/en-us/articles/360037225632-HaplotypeCaller). Variants were annotated using ANNOVAR (http://annovar.openbioinformatics.org/en/latest/), and Sanger sequencing was performed to confirm the variants. The gene variants were considered deleterious according to the following criteria: (1) absent from the GnomeAD and Exome Aggregation Consortium databases, (2) prediction tools (PolyPhen-2, SIFT, and MutationTaster) returned results favoring a deleterious effect on the gene, (3) gene mutations fit the disease-inheritance model, and (4) the phenotype of the patient was consistent with the gene-associated disease.

### Literature review

We reviewed relevant literature available in PubMed (https://pubmed.ncbi.nlm.nih.gov/) according to the following keywords: “*SLC39A14*,” “hypermanganesemia,” “manganism,” and “hypermanganesemia with dystonia.” The date of the last search was November 29, 2021. The case investigations included general clinical information about the patients, prominent neurological features, Mn levels, brain MRI results, gene mutations, treatments, and prognoses.

## Results

### Clinical description and investigation

The 10-month-old Chinese patient was conceived by in vitro fertilization, and his parents' ancestors, five generations ago, were from the same family. The bilateral fallopian tubes of the mother were obstructed, and in vitro fertilization and embryo transfer were performed twice. The first transplanted embryo was unviable at 30 days post-transplant, and the second transfer resulted in a twin, one of which stopped development at 7 weeks of gestation for unknown reasons, and the other one was the patient of the present case. The patient was born after 39 weeks of gestation by a cesarean section and weighed 2.99 kg. His length was 49 cm (the 95th percentile), and his head circumference was 37 cm (the 97th percentile). His postnatal period was uneventful.

The patient experienced delayed motor developmental milestones that included an inability to hold his head up and roll over at 4 months. He received rehabilitation with unremarkable outcomes. At 5 months of age, movement disorder presented along with rapidly progressive dystonia, bradykinesia, and spasticity. The GDS score was 72 points. He achieved a social smile at 2 months of age. At the time of evaluation for this study, he had slurred speech, and his visual and hearing abilities appeared normal. Significant environmental exposures to heavy metals and similar family history were ruled out.

A physical examination showed that his weight was 7 kg (the 10th percentile for age), and his head circumference was 41 cm (the 3rd percentile). He showed abnormal posture with his head tilted to the left side, which became more obvious when he was excited. He was unable to hold objects, as his thumbs were in an adducted position. The deep tendon reflexes were brisk with hypertonia. The Babinski signs were negative. There were no tremors or cranial nerve involvement, and systemic examination was normal.

Laboratory tests showed elevated Mn concentrations in the blood and urine [4.4 μg/dl (normal range: 0.4–1.4 μg/dl) and 1.2 μg/dl (normal range: <1 μg/dl), respectively], although normal ranges were detected in the parents. Other tests, including complete blood count, liver function, parathyroid hormone, and blood zinc, iron, cadmium, lead, and copper concentrations, were normal. Metabolic screening of plasma amino acids and urine organic acids was also normal.

**Figure 1:**
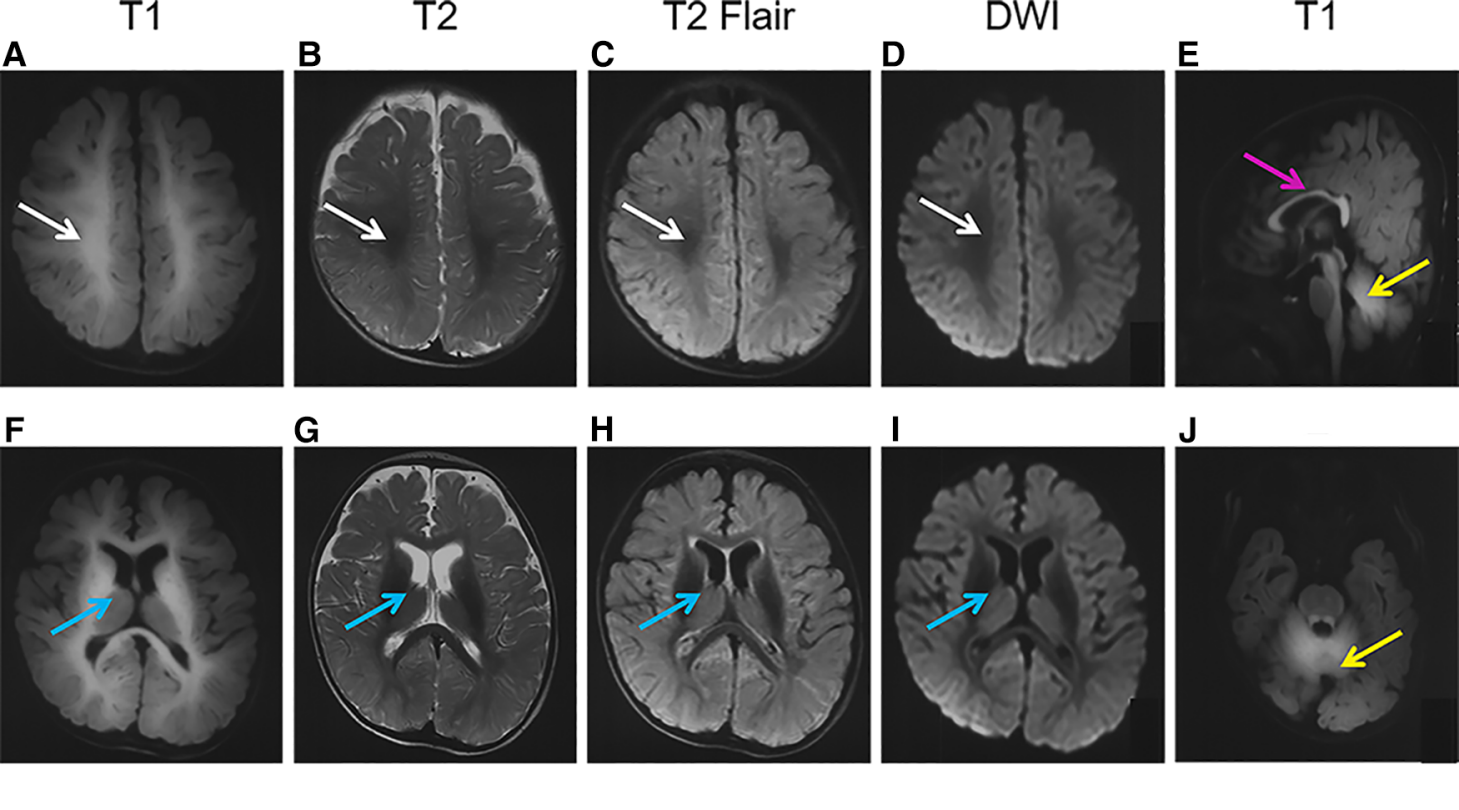
Brain magnetic resonance imaging (MRI) of the patient. The images show a high-T1 (**A,F,E,J**) and low-T2 (**B,G**), T2Flair (**C,H**), and DWI (**D,I**) signals in the regions of the centrum semiovale (**A–D**, white arrows), basal ganglia (F-I, blue arrows), corpus callosum (E, pink arrows), and cerebellum (**E,J**, yellow arrows).

Brain MRI performed at 7 months showed symmetrical hyperintensity on T1-weighted images and hypointensity on T2-weighted images, T2 fluid-attenuated inversion recovery (T2Flair), and diffusion-weighted imaging (DWI) in regions of the centrum semiovale, corpus callosum, internal capsule, cerebral peduncle, caudate, putamen, globus pallidus, dentate nuclei, and the deep white matter of the cerebrum and cerebellum (Figure 1).

The electroencephalogram (EEG) test was normal.

### Gene variants

**Figure 2:**
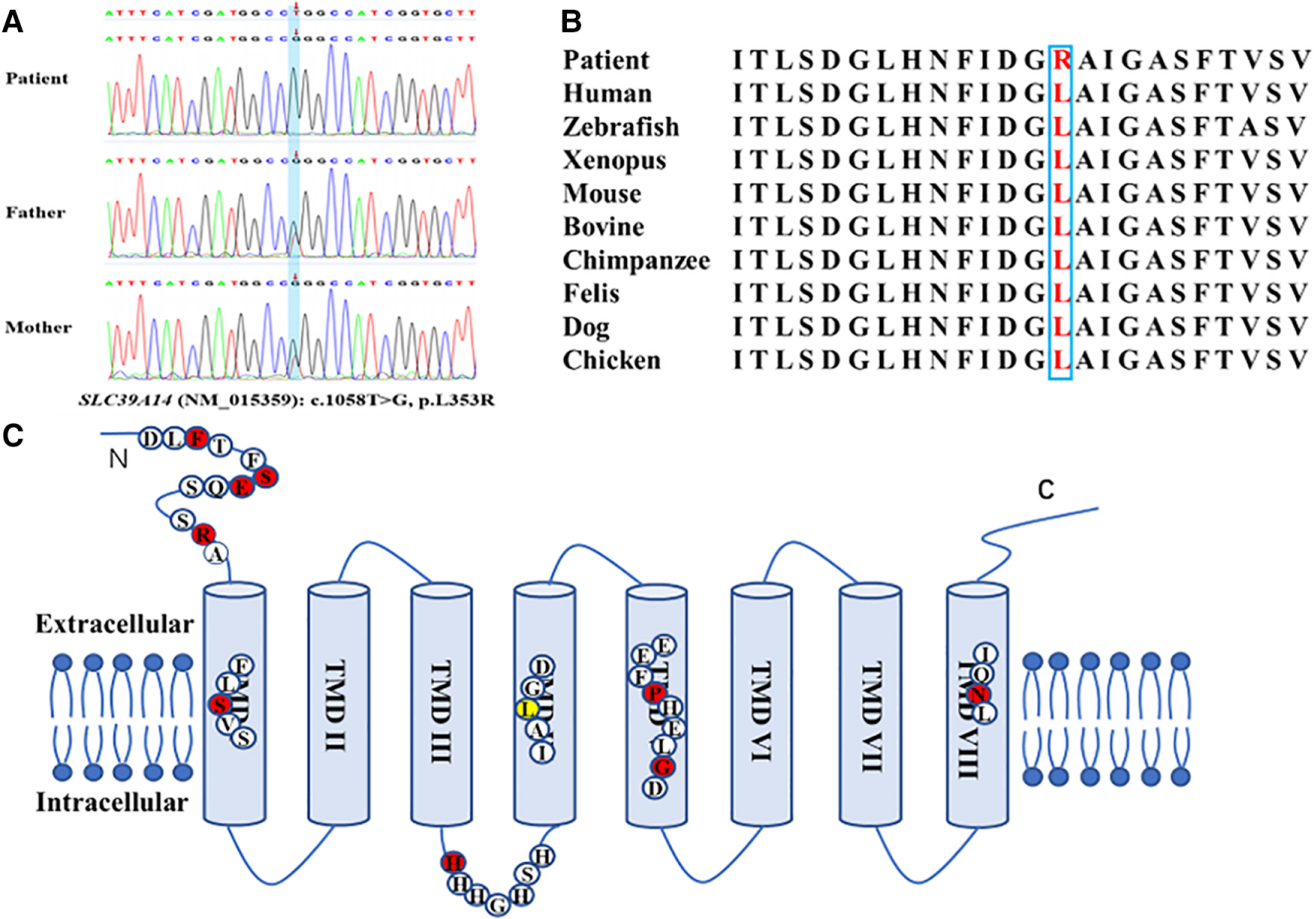
Novel homozygous variant of the *SLC39A14* gene identified in the patient. (**A**) Sequence chromatogram shows the homozygous variant of the *SLC39A14* gene in the patient and the heterozygous variant in both of his parents. (**B**) High conservation of the altered amino acid is shown in vertebrates and paralogous. (**C**) Simplified schematic and mutations ever identified in the *SLC39A14* gene. Red dot, mutations identified ever reported. Yellow dot, mutation identified in this study.

A novel homozygous variant of *SLC39A14* (c.1058T > G, p.L353R, NM_015359) was identified by WES in the patient, and a heterozygous variant was found in the parents (Figure 2A). This variant was located in the fourth transmembrane domain and affected a highly conserved amino acid residue in *SLC39A14* orthologs and paralogs (Figure 2B) but was not found in dbSNP, Human Gene Mutation Database, 1000 Genomes, and the Exome Aggregation Consortium. The mutation was predicted to be pathogenic by SIFT (https://faculty.washington.edu/wjs18/GS561/cSNPs_lab.html; 0.0), GERP++ (https://bio.tools/gerp#; 5.99), PROVEAN (http://provean.jcvi.org/index.php; −5.81), Mutation Taster (https://www.mutationtaster.org/; 1.0), and as possibly damaging by PolyPhen-2 (http://genetics.bwh.harvard.edu/pph2/; 0.996). The American College of Medical Genetics and Genomics scoring was PM2 + PP3 + PM3-Supporting, and the class was uncertain.

### Treatment and follow-up

Based on the clinical features and the gene mutation, the patient was diagnosed with HMNDYT2. A trial of Na_2_CaEDTA therapy (20 mg/kg/day in two divided doses for a 5-day course) was administered monthly at the age of 7 months. After 3 months of treatment, there was mild improvement in limb and neck dystonia to the extent that he could reach for objects with his right hand and achieve partial neck holding. The GDS score was 62 points, 10 points lower than before. Mn levels in the blood were reduced to 3.4 μg/dl but were not significantly altered in urine. The iron, zinc, cadmium, etc., concentrations in plasma were all normal. We observed slight decreases in abnormal signals in brain MRI results due to Mn deposition.

### Literature review

We identified five studies that included a total of 15 patients from 10 families, with consanguineous marriage in eight families (Table 1) (6, 8–11). These families were from different ethnic backgrounds, including Yemen, India, Lebanon, United Arab Emirates, Egypt, Senegal, Libya, and Spain (six families from Asia, three from Africa, and one from Europe). Among the 15 patients, 11 were girls and 4 were boys, and the mean age of onset was 14.9 months (range: 2–36 months). The initial clinical symptoms varied, with motor regression being the major complaint of 11 patients, followed by a delay of developmental milestones in two patients, clumsiness in writing in one patient, and paucity of movement of the right leg observed in one patient. During the disease course, all patients (except one not mentioned in the studies) lost ambulation before treatment. Fourteen patients developed progressive generalized dystonia, with eight reporting focal dystonia, as well (three had facial and five had oropharyngeal dystonia). Four patients presented parkinsonian features (hypomimia, bradykinesia, and tremor), and bulbar dysfunction was noted in six patients, spasticity in five, axial hypotonia in three, and hypokinesia in one patient. Four patients presented with hyperreflexia, whereas ankle clonus was present in three patients. Limb contracture occurred in four patients, and scoliosis occurred in three patients. Head circumference was described in five patients, of which four had microcephaly and one had macrocephaly.

**Table 1:** Clinical characteristics of the patients with *SLC39A14*-related mutations.

Subject	Sex	Country	Mutation and AA changed	Age of onset	Age of reporting	Age of death	Prominent neurological features	Abnormal head growth	Intelligence	Mn level	Brain MRI		Na_2_CaEDTA treatment			Other treatment
												Age at therapy	Dosage	Therapy duration	Clinical response	
I-1^ab^	F	Yemen	c.292T > G p.F98V	7 m	13 y		Severe neurological deterioration, spasticity, generalized and facial dystonia, loss of ambulation, hyperreflexia, ankle clonus	Microcephaly	Intellectual disability	2,887 nmol/L (blood) (NR 73–325 nmol/L)	T1 hyperintensity in cerebral and cerebellar white matter, globus pallidus, caudate, pituitary gland, dorsal pons; T2 and T2 Flair hypointensity in the globus pallidus; progressive cerebral atrophy	NA	NA	NA	NA	NA
I-2^ab^	F	Yemen	c.292T > G p.F98V	6 m	NA	8 y	Severe neurological deterioration, scant facial movements, spasticity, loss of ambulation, increased reflexes, ankle clonus	NA	Alert and responsive	NA	NA	NA	NA	NA	NA	NA
II-1^ab^	F	Egypt	NA	7 m	NA	13 m	Loss of acquired motor developmental milestones, dystonia, bulbar dysfunction, bradykinesia, axial hypotonia	NA	NA	NA	NA	NA	NA	NA	NA	NA
II-2^ab^	F	Egypt	c.313G > T p.E105X	7 m	3 y		Loss of acquired motor developmental milestones, dystonia, bulbar dysfunction, bradykinesia, axial hypotonia	NA	NA	8,101 nmol/L (blood) (NR 73–325 nmol/L)	T1 hyperintensity in cerebral and cerebellar white matter, globus pallidus, caudate, pituitary gland, dorsal pons and cerebellum; T2 and T2 Flair hypointensity in the globus pallidus	NA	NA	NA	NA	NA
III-1^ab^	F	India	c.477_478del p.S160Cfs*5	3 y	6 y		Clumsiness while writing and drawing, loss of the ability to stand and walk unaided, hypomimia, dystonia, hyperreflexia, ankle clonus, extensor plantar reflexes, tendon Achilles contractures	NA	normal	963 nmol/L (blood) (NR 73–325 nmol/L)	T1 hyperintensity in cerebral and cerebellar white matter, globus pallidus, caudate, pituitary gland, dorsal pons and cerebellum; T2 and T2 Flair hypointensity in the globus pallidus	5 y	500 mg twice daily for 5 consecutivedays	6 m	Upper limb tremors and athetoid movements had ceased and lower limb dystonia improved, walk independent-ly with foot orthoses	Baclofen, tetrabenazine, levodopa
IV-1^ab^	M	Spain	c.1147G > A p.G383R	10 m	NA	4 y	Motor developmental delay, dystonia, axial hypotonia, hypokinesia, lower limb hypertonia, opisthotonus	Macrocephaly	No language development	965 nmol/L (blood) (NR <145.6 nmol/L)	T1 hyperintensity in cerebral and cerebellar white matter, globus- pallidus, pituitary gland, dorsal pons and cerebellum; progressive cerebral atrophy	NA	NA	NA	NA	Levodopa, trihexyphenidyl, gabapentin and clonazepam
V-1^ab^	F	Lebanon	c.1407C > G p.N469K	2 y	17 y		Generalized and oromandibular dystonia, plantar flexion, loss of ambulation, limb contractures, scoliosis	Microcephaly	Learning disability	2,280 nmol/L (blood) (NR 73–325 nmol/L)	T1 hyperintensity in cerebral and cerebellar white matter, globus pallidus, caudate, pituitary gland, dorsal pons and cerebellum; T2 hypointensity in the globus pallidus	17 y	20 mg kg^−1^ per day	NA	Increase in urinary Mn excretion; reduction of blood Mn levels; deteriorate with worsening tremor and stiffness	Levodopa, benzodiazepines, trihexyphenid-yl, baclofen
V-2^ab^	F	Lebanon	c.1407C > G p.N469K	2 y	16 y		Generalized and oromandibular dystonia, plantar flexion, loss of ambulation, limb contractures, scoliosis	Microcephaly	Learning disability	3,830 nmol/L (blood) (NR 73–325 nmol/L)	T1 hyperintensity in cerebral and cerebellar white matter, globus pallidus, caudate, pituitary gland, dorsal pons and cerebellum; T2 hypointensity in the globus pallidus	NA	NA	NA	NA	Levodopa, benzodiazepines, trihexyphenidyl, baclofen
V-3^ab^	M	Lebanon	c.1407C > G p.N469K	2 y	9 y		Generalized and oromandibular dystonia, plantar flexion, loss of ambulation, limb contractures, scoliosis	Microcephaly	Learning disability	1,260 nmol/L (blood) (NR 73–325 nmol/L)	NA	NA	NA	NA	NA	Levodopa, benzodiazepines
VI-1^b^	F	India	c.382C > T p.R128W	2 m	1 y		Paucity of movement of right leg, dystonia, spasticity, inability to hold the head and roll over, difficulty in chewing, brisk deep tendon reflexes	Microcephaly	Intellectual disability	>3,640 nmol/L (blood) (NR 73–375 nmol/L)	T1 hyperintensity in cerebral and cerebellar white matter, globus pallidus, caudate, pituitary gland, dorsal pons and cerebellum; T2 hypointensity in the globus pallidus	1 y	20 mg/kg/dose for 5days	6 m	Reduction of blood Mn levels; improvement in swallowing and dystonia, achieved partial neck holding and corner sitting	Trihexiphenidyl
VII-1^ab^	M	Senegal	c.311G > T p.S104l	10 m	NA	21 m	Dystonic tetraparesis, rigidity, hypokinesia, pyramidal signs	NA	NA	NA	T1 hyperintensity in globus pallidal, cerebral and cerebellar white matter; T2 hypointensity in globus pallidal	NA	NA	NA	NA	NA
VII-2^ab^	M	Senegal	c.311G > T p.S104l	11 m	9 y		Generalized and oromandibular dystonia, neurological regression, opisthotonos, anarthria, dysphagia,	Microcephaly	NA	10.5 μg/L (Plasma) (NR 0.4–0.9 μg/L) 34 μg/L (CSF) (NR 0.5–1.7 μg/L) 8.2 μg/L (urine) (NR 0.4–0.9 μg/L)	T1 hyperintensity in globus pallidus, dentate nuclei, cerebral and cerebellar white matter; T2 hypointensity in globus pallidus, dentate nuclei; persistent cerebellar atrophy	9 y	1 g/m^2^/day in two divided oral doses	2 w	Worsening of cervical dystonia, irritability and sleep difficulties	
VIII-1^ab^	F	UAE	c.751-9C > G p.H251Pfs*26	8 m	9 y		Generalized and facial dystonia, dysarthric, difficulty in W-sit, crawling and feeding, inability to walk, action tremor	NA	Within the limits imposed by her motor impairment	64.2 μg/L (Serum) (NR4–16.5 μg/L)	T1 hyperintensity in basal ganglia, subthalamic nuclei, midbrain, pontine teg mentum, superior/middle cerebellar peduncles, cerebellar folia, dentate nuclei	5y	40 mg/kg/day, repeated 5-day cycles	4y	Increased urine Mn diuresis, serum Mn was not significantly altered; gradual disease progression	Dimercaptosuccinic acid, dpenicillamine, trihexyphenidyl, baclofen, clonazepam, L-dopa/carbidopa, Mn free diety
IX-1	F	UAE	c.751-9C > G p.H251Pfs*26	18 m	2 y		Have trouble walking, generalized and facial dystonia	NA	NA	78 μg/L (Serum) (NR4–16.5 μg/L)	T1 hyperintensity in globus pallidus, brainstem, cerebral and cerebellar white matter	NA	NA	1y	NA	NA
X-1^ab^	F	Libya	c.1136C > T p.P379L	30 m	3 y		Generalized dystonia, loss of ambulation, bulbar dysfunction	NA	NA	150 nmol/L (blood) (NR < 10 nmol/L)	T1 hyperintensity in brain stem, basal ganglia; T2 hypointensity in globus pallidus	NA	NA	Just started	NA	NA
Our Study^ab^	M	China	c.1058T > G p.L353R	4 m	10 m		Delayed motor developmental milestones, generalized dystonia, bradykinesia, spasticity, hyperreflexia	Microcephaly	Intellectual disability	4.4 μg/dL (blood) (NR 0.4–1.4 μg/dL) 1.2 μg/dL (urine) (NR < 1μg/dL)	T1 hyperintensity and T2, T2 Flair, DWI hypointensity in centrum semiovale, corpus callosum, internal capsule, cerebral peduncle, caudate, putamen, globus pallidus, dentate nuclei, and the deep white matter of the cerebrum and cerebellum	7 m	20 mg/kg/day in two divided dose for a 5-day course	3 m	Mild improvement in limb and neck dystonia, reduction of blood Mn levels	No

F, female; M, male; Mn, manganese; NA, not available; NR, normal range; UAE, United Arab Emirates; m, months; w, weeks; y, years.

^a^ Patients from consanguineous families.

^b^ Both parents are heterozygous carriers of the identified mutation.

Normal cognitive function was reported in one patient, whereas three patients showed intelligence within limits imposed by motor impairment, and three showed mild impairment with a degree of learning disability. Two patients presented with intellectual disabilities, and one presented with no language development. The intellectual development of the other patients was not reported.

Among the 15 patients, 12 had the results of Mn levels. All of them presented hypermanganesemia, with values 4–25 times higher than the normal. Additionally, one patient (VII-2) showed elevated Mn levels in cerebrospinal fluid (34 μg/L; normal range: 0.5–1.7 μg/L) and urine (8.2 μg/L; normal range: 0.4–0.9 μg/L).

Brain MRIs of the 12 patients revealed that all patients showed T1-weighted hyperintensity in the basal ganglia, especially in the globus pallidus. T1 hyperintensity was also noted in the cerebral and cerebellar white matter in 10 patients, the pituitary gland and dorsal pons in 7 patients, the brain stem in 3 patients, and the dentate nucleus in 2 patients. T2 hypointensity in the globus pallidus was reported in nine patients. There was no description of T2Flair or DWI values in these patients. Progressive cerebral atrophy was reported in three patients.

Gene mutation tests were performed on 14 patients from 10 families and 9 parents, with definite homozygous mutations of *SLC39A14* identified in all patients and heterozygous mutations in all parents. The same mutation was found in two unrelated families from the United Arab Emirates, resulting in a total of nine identified mutations, including six missense mutations [c.292T > G (p.F98V), c.1147G > A (p.G383R), c.1407C > G (p.N469K), c.382C > T (R128W), c.311G > T (p.S104I), and c.1136C > T (p.P379L)], one intronic aberrant-splicing mutation [c.751-9C > G (H251Pfs*26)], one nonsense mutation [c.313G > T (p.E105X)], and one deletion mutation [c.477_478del (p.S160Cfs*5)], among the ten families (Figure 2C). The mutation was not reported in patient II-1.

Seven patients were treated with Na_2_CaEDTA at ages ranging from 1 to 17 years for 2 weeks to 4 years. Patient III-1 initiated treatment (500 mg twice daily for 5 consecutive days) at 5 years of age, which resulted in an obvious clinical improvement that included walking ability after 6 months of monthly therapy. Patients V-1, VI-1, and VII-2 initiated treatment at ages 17, 1, and 9 years, respectively, at dosages ranging from 20 mg/kg/day iv to 1 g/m^2^/day in two oral doses for 5 days every month. All three patients showed reduced blood Mn levels; however, the clinical improvement was unremarkable or continued to deteriorate. Patient VIII-1 was treated with 40 mg/kg/day Na_2_CaEDTA in 5-day cycles every month from age 5 to 9 years, during which Mn excretion in urine increased in the absence of significant changes in serum Mn levels. Additionally, this patient failed to show clinical response to two oral chelators (2,3-dimercaptosuccinic acid and D-penicillamine), and at age 8 years, she maintained “Mn-free diet” for 2–3 days per week. Despite various treatment options, the disease course continued to deteriorate. Patient IX-1 received chelation therapy for 1 year and then gave up. Patient X-1 had recently initiated chelation therapy according to the study; therefore, the effectiveness was unknown. Eight patients underwent a clinical trial with levodopa, trihexyphenidyl, baclofen, or benzodiazepines that resulted in no or minimal improvement in symptoms.

Four patients died between ages 13 months and 7 years, with two dying of respiratory tract infections and causes of death unknown for the remaining.

## Discussion

Mn is an important trace element for several metabolic pathways; however, an excessive amount in the body is neurotoxic. To date, three inborn errors of Mn homeostasis have been identified (12). One is referred to as hypermanganesemia with dystonia, polycythemia, and cirrhosis (HMNDYT1, MIM 613280), which is caused by mutations in the *SLC30A10* gene (7, 13). *SLC30A10* encodes a divalent cation transporter that localizes to the cell membrane and mediates Mn biliary excretion (14). Another is a congenital disorder of glycosylation type IIn (*SLC39A8*-CDG, MIM 616721), caused by *SLC39A8* gene mutations. *SLC39A8* encodes an Mn-uptake transporter that mediates Mn influx. The major clinical manifestations of this disorder include developmental delay, intellectual disability, short stature, seizures, cerebellar atrophy, and Leigh-like syndrome (15). The third disorder is HMNDYT2 caused by *SLC39A14* mutation (6).

*SLC39A14* [also known as Zrt-, Irt-like protein 14 (ZIP14)] is a member of the LIV-1 family and encodes a divalent cation transporter expressed on the cell membrane. Initially, it was considered an important protein for zinc transport (16); however, subsequent studies confirmed its roles in transporting iron and cadmium (17–19). In 2016, Tuschl et al. first reported a cohort of children with hypermanganesemia and progressive parkinsonism–dystonia and who carry homozygous mutations in *SLC39A14* (6, 19). This discovery demonstrated that *SLC39A14* also regulates Mn homeostasis; however, to date, all patients reportedly carrying *SLC39A14* mutations (including the patient in the present study) developed hypermanganesemia, whereas blood levels of zinc, iron, and cadmium remained within the normal range (6, 8–11). These findings indicate that *SLC39A14* functions as a pivotal Mn transporter and that associated mutations impair Mn elimination without affecting the homeostasis of other mental ions. It was also confirmed in zebrafish with CRISPR-induced *slc39a14* null mutations (6).

Although Mn requires close regulation, the mechanisms associated with Mn homeostasis remain unknown. Loss-of-function experiments in zebrafish by Tuschl et al. showed that *SLC39A14* is involved in hepatocyte uptake of Mn from the circulation for biliary excretion (6); however, loss of *SLC39A14* expression selectively in hepatocytes was not sufficient to cause Mn accumulation (20, 21). Additionally, intestine-specific knockout of Zip14 in mice resulted in increased Mn levels in both the liver and brain under normal dietary conditions, suggesting a role for *SLC39A14* in limiting Mn absorption and that absence of *SLC39A14* in the intestine increases Mn absorption to induce Mn overload (20, 22). These findings suggest that limiting intestinal *SLC39A14*-mediated Mn absorption and increasing hepatic *SLC39A14*-mediated clearance from the portal blood cooperatively regulates Mn homeostasis in humans (23).

HMNDYT2 caused by *SLC39A14* mutation is a very rare disease. To date, only 16 patients (including that in the present study) from 11 families have been reported worldwide (Table 1) (6, 8–11). Among them, 14 cases were from 9 consanguineous families, and the highest incidence is in Asia (10 cases). Females are more likely to be affected (11 females and 5 males); this is consistent with Indian patients (24). However, it is impossible to speculate on a relationship between gender, race, and incidence according to the small number of cases. It is worth noting that the clinical features of all cases were highly similar. Affected children usually present with motor regression or delay of developmental milestones in infancy or early childhood, followed by the presentation of generalized dystonia, loss of ambulation, spasticity, parkinsonian features, and so on. In contrast to motor disability, there appears to be a degree of cognition sparing affected. The cognitive development may be related to the age of onset. Previous studies report 3 years as the onset age in one patient with normal intelligence, whereas in two cases with mental retardation, onset occurred at 2 and 7 months, respectively. This suggests that earlier age of onset results in more severe cognitive impairment. Moreover, both microcephaly and macrocephaly have been observed in this disorder.

All cases reporting Mn levels presented with hypermanganesemia, with values 4–25 times higher than normal, although no significant correlation has been identified between disease severity and blood Mn levels. The youngest affected child (onset at 2 months) showed blood Mn levels 10 times higher than normal but also presented normal early motor development. By contrast, the patient in the present study showed no motor development milestones and blood Mn levels only threefold higher than normal. Therefore, a relationship between blood Mn levels and clinical severity requires further investigation.

As a mineral deposition disease, the characteristics of the brain image were specific. On brain MRI (Figure 1) of the patient in the present study, deposition of Mn is evident in the basal ganglia, particularly the globus pallidus and striatum, as well as the regions of centrum semiovale and corpus callosum, with pronounced hyperintensity on T1-weighted imaging and corresponding hypointensity on T2-weighted imaging, as well as on T2Flair and DWI. The abnormal signals in the brain MRI got better after 3 months of treatment. Such results had not been reported previously, and continual follow-up was needed.

The rate of *SLC39A14* mutations leading to HMNDYT2 is very low in the population. To date, only 10 variants from 11 families (including that in the present study) have been detected worldwide, of which 9 were from consanguineous families. This reveals that *SLC39A14* is a highly conserved gene. As a divalent metal transporter, *SLC39A14* contains eight transmembrane domains (TMDs) with a long extracellular N-terminus and a short extracellular C-terminus (6). It is distinguished by the consensus motif (H/EEXPHEXGD) in TMD V and a histidine-rich motif (HXHXHX) in the loop between TMD III and TMD IV (Figure 2C) (25). There are five mutations located in TMDs I, IV, V, and VIII. TMDs IV–VIII were found to be the greatest degree of conservation in the Zip family (23, 24). The frameshift mutation (S160Cfs*5) reported by Tuschl et al. is located in TMD I and likely results in significantly truncated proteins (3). The c.1058T > G (p.L353R) variant found in the patient in the present study is located in TMD IV, affects a conserved amino acid residue, and represents a novel variant not previously reported. Moreover, the mutation was predicted as deleterious by bioinformatics methods, suggesting that it is likely responsible for the hypermanganesemia and neurodegeneration observed in this patient. Previous studies report that the c.751-9C > G (p.H251Pfs*26) variant represents the only mutation occurring in two unrelated families from the United Arab Emirates (10). This mutation is located within the histidine-rich motif, and aberrant splicing results in a stop codon. Additionally, the conserved extracellular domain in the N-terminus seems to be a region prone to mutate. Four variants from six patients are located in this region and reportedly alter protein folding and trafficking, leading to pathogenicity (6, 8, 9, 26). Patients harboring these mutations demonstrate an earlier onset age (range: 2–11 months). Larger-scale studies are needed to identify the genotype–phenotype relationship.

Na_2_CaEDTA is a chelating agent that promotes the aggregation of metal ions into stable and soluble complexes that allow their excretion in the urine. Treatment with this chelator effectively reduces Mn accumulation, ameliorates neurological symptoms, and prevents liver disease progression in patients harboring *SLC30A10* mutations (27). For *SLC39A14*-associated hypermanganesemia, the clinical response to Na_2_CaEDTA treatment varies. It seems that the later onset or earlier treatment may have better outcomes; however, there are no significant correlations between treatment duration and clinical response. Further treatment studies with larger sample sizes and longer periods are needed.

In addition to Mn chelation, a dietary restriction was suspected to be beneficial for the reduction of Mn levels and improvement of neurologic symptoms. However, Patient VIII-1 received 2–3 days of Mn free per week and Na_2_CaEDTA therapy simultaneously, and no obvious improvement in clinical symptoms was achieved. As Mn is ubiquitous in food, formula with an Mn-free or low-Mn diet is challenging. For the symptoms of dystonia and parkinsonian features, some drugs such as levodopa, trihexyphenidyl, and so on were trialed and proved to have little clinical benefit in the literature.

As a rare disease, HMNDYT2 usually occurs in consanguineous families and represents a rapidly progressing disease with a poor prognosis. Na_2_CaEDTA appears to effectively chelate Mn and increase urinary Mn excretion in affected individuals; however, the clinical response varies. Studies suggest that chelation therapy might be more effective in preventing disease progression if initiated earlier. Early diagnosis is critical to the initiation of appropriate treatment. This report expands the genotype spectrum of this rare disease. However, more effective treatments still need to be explored.

## Data Availability

The original contributions presented in the study are included in the article/Supplementary Material; further inquiries can be directed to the corresponding author/s.
